# Expression of citrulline and homocitrulline residues in the lungs of non-smokers and smokers: implications for autoimmunity in rheumatoid arthritis

**DOI:** 10.1186/s13075-015-0520-x

**Published:** 2015-01-20

**Authors:** Elena B Lugli, Raquel ESM Correia, Roman Fischer, Karin Lundberg, Ken R Bracke, Anna B Montgomery, Benedikt M Kessler, Guy G Brusselle, Patrick J Venables

**Affiliations:** Kennedy Institute of Rheumatology, University of Oxford, Roosevelt Drive, Headington, Oxford OX3 7FY UK; Target Discovery Institute, Nuffield Department of Medicine, University of Oxford, Old Road Campus, Headington, Oxford OX3 7FZ UK; Karolinska Institute, Department of Medicine, Solnavägen 1, Solna, 171 76 Stockholm Sweden; Laboratory for Translational Research of Obstructive Pulmonary Disease, Department of Respiratory Medicine, Ghent University Hospital, De Pintelaan, 185-9000 Ghent, Belgium

## Abstract

**Introduction:**

Smoking is a well-established risk factor for rheumatoid arthritis (RA), and it has been proposed that smoking-induced citrullination renders autoantigens immunogenic. To investigate this mechanism, we examined human lung tissue from 40 subjects with defined smoking status, with or without chronic obstructive pulmonary disease (COPD), and control tissues from other organs for citrullinated proteins and the deiminating enzymes peptidylarginine deiminase type-2 (PAD2) and -4 (PAD4).

**Methods:**

Lung tissue samples, dissected from lobectomy specimens from 10 never smokers, 10 smokers without airflow limitation, 13 COPD smokers and eight COPD ex-smokers, and control tissue samples (spleen, skeletal muscle, liver, ovary, lymph node, kidney and heart), were analysed for citrullinated proteins, PAD2 and PAD4 by immunoblotting. Citrulline and homocitrulline residues in enolase and vimentin were analysed by partial purification by gel electrophoresis followed by mass spectrometry in 12 of the lung samples and one from each control tissues. Band intensities were scored semi-quantitatively and analysed by two-tailed Mann-Whitney T-test.

**Results:**

Within the lung tissue samples, citrullinated proteins, PAD2 and PAD4 were found in all samples, with an increase in citrullination in COPD (*P* = 0.039), but minimal difference between smokers and non-smokers (*P* = 0.77). Citrullination was also detected at lower levels in the tissues from other organs, principally in lymph node, kidney and skeletal muscle. Mass spectrometry of the lung samples showed that vimentin was citrullinated at positions 71, 304, 346, 410 and 450 in non-smokers and smokers both with and without COPD. A homocitrulline at position 104 was found in four out of six COPD samples and one out of six non-COPD. Citrulline-450 was also found in three of the control tissues. There were no citrulline or homocitrulline residues demonstrated in α-enolase.

**Conclusions:**

We have shown evidence of citrullination of vimentin, a major autoantigen in RA, in both non-smokers and smokers. The increase in citrullinated proteins in COPD suggests that citrullination in the lungs of smokers is mainly due to inflammation. The ubiquity of citrullination of vimentin in the lungs and other tissues suggests that the relationship between smoking and autoimmunity in RA may be more complex than previously thought.

**Electronic supplementary material:**

The online version of this article (doi:10.1186/s13075-015-0520-x) contains supplementary material, which is available to authorized users.

## Introduction

Rheumatoid arthritis (RA) is a true autoimmune disease driven by anti-citrullinated peptide/protein antibodies (ACPA) [1]. ACPA may be detected in RA serum more than 10 years before onset of disease, suggesting that the autoimmune response begins outside the joint [2]. In established disease, ACPA are strongly associated with smoking and the shared epitope, with antibodies to citrullinated α-enolase peptide 1 (CEP-1) and citrullinated vimentin giving the highest odds ratios [3]. This suggests that citrullinated enolase and vimentin in the lung could prime the autoantibody response.

It is widely thought that alterations in protein structure and antigenicity, resulting from the post-translational modification of arginine to citrulline, may break tolerance to citrullinated proteins and drive an autoantibody response, which eventually leads to disease [1,4]. Citrullination is mediated by peptidylarginine deiminases (PADs), of which PAD2 and PAD4 are likely to be most important in RA [5]. Because smoking is a risk factor for RA [6,7], it has been suggested that smoke inhalation may increase the concentration of the PADs, which in turn leads to increased formation of citrullinated proteins driving an ACPA response. Although this is a simple and widely quoted hypothesis, the evidence in its support is derived from one study in which bronchoalveolar lavage (BAL) cells showed a modest increase in the concentration of PAD2 and citrullinated proteins from smokers [8]. However, the same study showed constitutive expression of citrullinated proteins within bronchial mucosal biopsy samples in healthy lung tissue from both smokers and non-smokers.

In this study, we investigated surgically excised samples of human lung from patients without RA (n = 41), in whom smoking history and presence or absence of chronic obstructive pulmonary disease (COPD) were accurately documented (Additional file 1: Table S1). The documentation of COPD is important, because pulmonary inflammation is amplified [9], and persists despite smoking cessation [10]. To test the organ-specificity of citrullination, we have also analysed surgically excised samples of other human tissues (lymph node, liver, kidney, spleen, ovary, heart and skeletal muscle, n = 24). The samples were from surgery providing sufficient material for immunoblotting [9], which allows for more specificity when using some of the available antibodies to PAD2 and PAD4 [11], and the anti-modified citrulline (AMC) kit for examining citrullinated proteins. By these means we have examined the expression of citrullinated antigens and the PAD enzymes, and their relationship to COPD and smoking.

## Methods

### Lung samples

Lung tissue was carefully excised from lobectomy specimens removed from patients diagnosed with solitary pulmonary lesions, mostly lung tumours (n = 41). Only uninvolved lung tissue, at maximal distance from the lung tumour, has been used for the current study. All patients attended Ghent University Hospital and samples were donated with informed consent and approval from the Medical Ethics Committee (Belgium registration number: B670201110668). Based on spirometry, according to the Global Initiative for Chronic Obstructive Lung Disease (GOLD) guidelines, and smoking history, patients were divided into four groups: smokers with (n = 13) and without (n = 10) COPD, ex-smokers with COPD (n = 8) and never smokers without COPD (n = 10). For details of the study subjects see Additional file 1: Table S1. The lung tissue was homogenised in RIPA buffer with protease inhibitors, centrifuged and the supernatants used for further experiments.

### Control tissue samples from other organs

Uninvolved surgically removed human tissue samples (spleen (n = 5), skeletal muscle (n = 2), liver (n = 4), ovary (n = 4), lymph node (n = 4), kidney (n = 4) and heart (n = 1)) were provided by Imperial College Healthcare NHS Trust Tissue Bank with appropriate informed consent and ethical approval (Imperial College Healthcare Tissue Bank HTA licence: 12275; and Research Ethics Committee Wales approval: 12/WA/0196). These control tissues were homogenised as described above.

### Immunoblotting and quantification

Lung and control tissue lysates (20 μg/sample) were resolved on 4 to 12% Bis-Tris NUPAGE gels (Invitrogen, Carlsbad, CA, USA), transferred to nitrocellulose membranes (Invitrogen) and western blotted using a standard protocol.

The following primary antibodies were used: rabbit anti-fibrinogen (1:5,000; Calbiochem, San Diego, CA, USA), rabbit anti-PAD2 (1:500; Abcam, Cambridge, UK), goat anti-PAD4 (1:800; Abcam), rabbit anti-vimentin (1:1,000; Santa Cruz Biotechnology, Dallas, TX, USA), rabbit anti-CEP1 (1:10,000; in house,[12]) and AMC antibody (Merck Millipore, Darmstadt, Germany, according to the manufacturer’s instructions). All secondary antibodies were horseradish peroxidase (HRP)-conjugated and diluted 1:5,000.

Recombinant PAD2, PAD4, and *in vitro* citrullinated recombinant α-enolase were used as positive controls where needed.

Band intensities were quantified semi-quantitatively by three independent individuals using a four point (0 to 3) scoring method. The mean and median scores were analysed by statistical software GraphPad Prism (GraphPad Software, La Jolla, CA, USA).

### Statistics

Statistical differences between intensity scores in each lung group were calculated by either one-way analysis of variance (ANOVA) when comparing all groups together or by two-tailed Mann-Whitney T-test using GraphPad Prism software. A value of *P* <0.05 was considered significant.

### Mass spectrometry

Representative tissue lysates from 12 lung samples and one sample from each of the control tissues were selected according to their levels of citrullination on the AMC blots. A 5 mm band in the region of 52 kiloDaltons (kDa) was excised from a Coomassie-stained gel and used for mass spectrometry analysis. Sample preparation was performed as described previously [13]. Briefly, proteins were reduced with dithiothreitol and alkylated with iodoacetamide before being digested with trypsin (Promega, Madison, WI, USA) and injected into a nLC-MC/MC workflow (Q Exactive mass spectrometer coupled with a Dionex Ultimate 3000 UPLC; Dionex, Sunnyvale, CA, USA). Samples were desalted online (PepMAP C18, 300 μm × 5 mm, 5 μm particle; Thermo Fisher Scientific, Waltham, MA, USA) for 1 minute at a flow rate of 20 μl/min and separated on a nEASY column (PepMAP C18, 75 μm × 500 mm, 2 μm particle; Thermo Fisher Scientific) over 60 minutes using a gradient of 2% to 35% acetonitrile 0.1% acid at 250 nl/min. Survey scans were acquired at a resolution of 70,000 at 200 m/z and the 15 most abundant precursors were selected for high-energy collision dissociation (HCD) fragmentation. Mass spectrometry data was analysed in Peaks (V.6; Bioinformatics Solutions, Waterloo, ON, Canada) using the post-translational modification search option at mass tolerances of 10 ppm for MS1 and 0.05 Da for MS2 at 1% false discovery rate (FDR). Citrullinated peptides were manually inspected when the citrullinated residue produced a missed cleavage to exclude false positive assignment of citrullination (that is deamidation or the selection of the first isotopic peak as precursor mass).

## Results

### Citrullinated proteins are abundantly expressed in lung tissue

**Figure 1:**
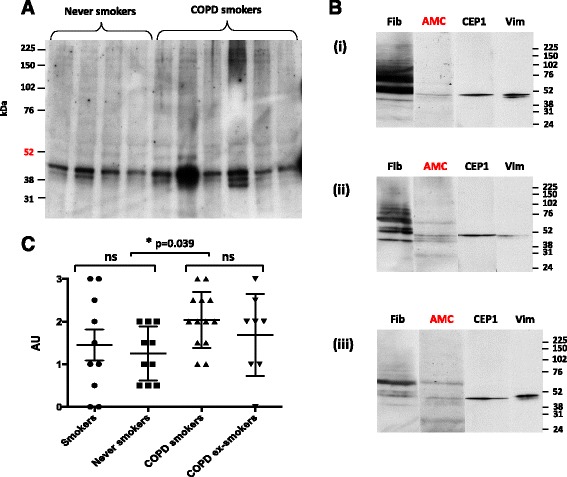
**Citrullinated proteins are found in all lung tissue lysates. (A)** A representative immunoblot with the AMC antibody of proteins (20 μg/lane) from lung tissue lysates of four never smokers and six COPD smokers. **(B)** One lung sample from each patient group was selected and run on a single-welled SDS-PAGE gel. Each blot was cut into four strips that were used to determine presence of specific proteins (fibrinogen (Fib), citrullinated proteins (AMC), CEP-1 and vimentin (Vim)) with their corresponding antibodies. The immunoblot strips for each lung sample were aligned with the molecular weight markers (kDa). The results shown are three representative blots for a (i) non-smoker, (ii) COPD smoker, (iii) smoker without COPD. **(C)** The intensity of citrullinated proteins in each lung sample was assessed by blinded semi-quantitative analysis using a 4-point scale (0 to 3) (arbitrary units (AU)). The bar diagrams represent mean ± standard error of the mean (SEM); NS, not statistically significant. AMC, anti-modified citrulline; CEP-1, citrullinated α-enolase peptide 1; COPD, chronic obstructive pulmonary disease; kDa, kiloDaltons.

**Figure 2:**
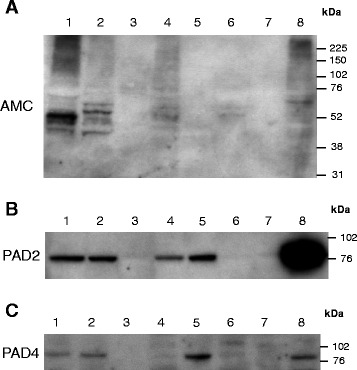
**Citrullination occurs in several human organs.** Representative immunoblot with **(A)** AMC, **(B)** anti-PAD2 and **(C)** anti-PAD4 antibodies on proteins (20 μg/lane) from one COPD lung tissue lysate (lane 1) and control tissue lysates from lymph node (lane 2), liver (lane 3), kidney (lane 4), spleen (lane 5), ovary (lane 6), heart (lane 7) and skeletal muscle (lane 8). AMC, anti-modified citrulline; COPD, chronic obstructive pulmonary disease; PAD, peptidylarginine deiminase.

Using the AMC antibody, citrullinated proteins were detected in all lung samples examined (Figure 1A). There were prominent bands near the 52 kDa marker, which co-migrated with polypeptides representing enolase, vimentin and part of fibrinogen (Figure 1B). The level of citrullination from the AMC blots was scored semi-quantitatively and showed that the patients with COPD (current smokers and ex-smokers) had median scores of 2.0 compared with the non-COPD (smokers and never smokers) who had median scores of 1.25. This difference between the COPD groups and the non-COPD groups reached statistical significance (*P* = 0.039), though there was less difference between the smokers and non-smokers combining COPD and non-COPD (mean score 1.90 vs 1.35; *P* = 0.46 vs *P* = 0.77) (Figure 1C). This suggests that the increase in citrullination seen in the COPD groups was more related to COPD itself, rather than to smoking. When analysing other human tissues using the AMC antibody, we detected the presence of citrullination in some of the tissues, but with much lower intensity compared with the lung samples (Figure 2A), suggesting, amongst the tissues examined, that the lung is more prone to citrullination.

### PAD2, PAD4 and the RA antigens vimentin, fibrinogen and enolase are present in lung tissue

**Figure 3:**
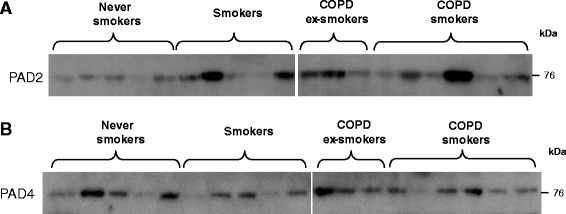
**PAD2 and PAD4 are present in all lung tissues.** Representative immunoblots with **(A)** anti-PAD2, and **(B)** anti-PAD4 of lung lysates (20 μg/lane) from never smokers (n = 5), smokers without COPD (n = 5), COPD smokers (n = 6) and COPD ex-smokers (n = 3). COPD, chronic obstructive pulmonary disease; PAD, peptidylarginine deiminase.

PAD2 and PAD4 were mainly found in the lung, lymph node, spleen and skeletal muscle, unrelated with the amount of citrullination in each corresponding sample (Figures 2 and 3). Both enzymes were found in all of the lung samples, with a marginal increase of PAD2 in the smokers (Figure 3). However, this did not reach statistical significance.

In the absence of commercially available antibodies to citrullinated vimentin and fibrinogen, we used antibodies to the native proteins, which also react with citrullinated forms of the proteins. For enolase, we have used an antibody raised against the CEP-1 peptide, which reacts 100-fold more sensitively with citrullinated enolase than with the native molecule [11]. As expected, vimentin, as a ubiquitous cellular protein, and fibrinogen, as a prominent protein in serum and extracellular fluid, were detected in all of the samples, as was enolase, as a highly multifunctional conserved protein.

### Mass spectrometry to determine the dominant citrullinated proteins in the lung

Representative tissue lysates were selected from 12 of the lung samples and one sample from each of the other tissues for characterisation of RA antigens by mass spectrometry. Citrullinated peptides were inspected for validity of site assignment (Additional file 2: Figures S1-S7).

Gel slices from an approximate 50 kDa region were excised for digestion and peptide sequencing, because this was the area of maximum citrullination (Figure 1B). In this region α-enolase and vimentin were the two most abundant proteins identified. Sequence coverage for vimentin was 97% and 95% for α-enolase in the combined samples. We identified five citrullinated vimentin peptides with citrullines at positions 71, 304, 346, 410 and 450 in the lung samples from the non-smokers and smokers with and without COPD (Table 1 and Additional file 2: Figures S1-S6). We detected two citrullinated vimentin peptides in other human tissues (citrulline at positions 304 and 450), both shared with the lung samples (Table 1). Citrulline-304 was found in kidney, ovary and spleen and citrulline-450 in kidney, ovary and lymph node. Alpha enolase peptides were only detected in their native non-citrullinated form including arginines at positions 9 and 15 corresponding to the citrulline residues in the immunodominant CEP-1 peptide [12].

**Table 1:** **Citrullinated (cit) and homocitrullinated (Hcit) vimentin peptides identified in human tissue samples**

**Vimentin peptides**		**Frequency of detection**										
		**Lung**				**Lymph node**	**Liver**	**Kidney**	**Spleen**	**Ovary**	**Heart**	**Skeletal muscle**
		**COPD smokers**	**COPD ex-smokers**	**Never smokers**	**Smokers without COPD**							
Citrullinated	_70_ L(cit)SSVPGVR_78_ / _71_(cit)SSVPGVR_78_	2/4	1/2	2/3	2/3	0/1	0/1	0/1	0/1	0/1	0/1	0/1
	_295_FADLSEAAN(cit)NNDALR_310_	0/4	0/2	1/3	0/3	0/1	0/1	1/1	1/1	1/1	0/1	0/1
	_344_QM(cit)EMEENFAVEAANYQDTIGR_365_	0/4	0/2	1/3	0/3	0/1	0/1	0/1	0/1	0/1	0/1	0/1
	_403_LLEGEES(cit)ISLPLPNFSSLNLR_424_	1/4	0/2	1/3	1/3	0/1	0/1	0/1	0/1	0/1	0/1	0/1
	_446_TVET(cit)DGQVINETSQHHDDLE_466_	1/4	0/2	2/3	2/3	1/1	0/1	1/1	0/1	1/1	0/1	0/1
Homocitrullinated	_101_TNE(Hcit)VELQELNDRFANYIDKVR_122_	2/4	2/2	1/3	0/3	0/1	0/1	0/1	0/1	0/1	0/1	0/1

Representative tissue lysates from 12 lung tissues (COPD smokers (n = 4), COPD ex-smokers (n = 2), never smokers (n = 3) and smokers without COPD (n = 3)) and one from each of the control tissues were analysed by mass spectrometry. COPD, chronic obstructive pulmonary disease.

We also detected one vimentin peptide with a homocitrulline residue (Table 1 and Additional file 2: Figure S7) in four of six COPD samples and in one of the never smokers (one out of three). No homocitrullines were detected in α-enolase.

## Discussion

We have shown the expression of citrullinated proteins and the presence of citrullinating enzymes PAD2 and PAD4 in lung tissue of both smokers and non-smokers. Lower levels of citrullination were seen in lymph node, kidney and skeletal muscle tissues. Like previous reports using immunohistochemistry [8], we found increased presence of PAD2 and citrullination in the smokers, but in our study, this was mainly associated with COPD rather than smoking itself. Given that pulmonary inflammation is amplified in patients with COPD, our study highlights inflammation in the lung as a likely basis for epidemiological associations between smoking and the ACPA response in RA.

The importance of inflammation rather than smoking itself is supported by the finding that bronchiectasis, one of the most severe infectious disease of the lung, is also a risk factor for ACPA-positive RA, and that this risk is independent of smoking [14,15]. Citrullination resulting from inflammation has also been reported in organs other than the lungs. Indeed, citrullination detected by immunohistochemistry in synovium, muscle and colon was roughly in proportion to the amount of inflammation present in these tissues [16]. Periodontitis, often cited the commonest chronic infectious disease of humans, is another smoking-independent environmental risk factor for RA [17], and shows that the inciting inflammatory response does not necessarily have to begin in the lung.

A potential criticism of our study is that the pathology for which the lobectomies were performed might have influenced the citrullination in the samples examined. Most of the samples were from patients with lung tumours, which being judged as resectable, were focal, not treated with chemotherapy, surrounded by a good margin of unaffected tissue [9] and with the study sample taken as far away from the primary lesion as possible. Moreover, it is well known that citrullination associated with inflammation usually occurs immediately adjacent to inflammatory infiltrates [16]. Therefore, we can conclude that the pathology distant from the sample of lung examined is unlikely to have affected the results in our study.

Using mass spectrometry, we found that vimentin was citrullinated at positions 71, 304, 346, 410 and 450 in non-smokers and in smokers with and without COPD. A lower frequency of citrullinated vimentin peptides (citrulline-450 and -304) was found in other human tissues, which suggests that citrullination of vimentin is ubiquitous in healthy tissues. A recent report also found citrulline-450 to be expressed in both the lungs and the synovial membrane of patients with RA [18] and it was suggested that it represented a shared immunological target between the lungs and the joints. Our data does not contradict this conclusion, but does show that the lungs and the joints are not unique in this respect. The ubiquity of citrulline-450 also questions how important smoking is in relation to the expression of this residue.

Another citrullinated residue in our study, citrulline-71, has not been previously described in lung tissue. Like citrulline-450 we found it to be apparently independent of both smoking and COPD, though firm conclusions must be tempered by the small number of tissue samples examined. Unlike citrulline-450, we did not find citrulline-71 in any of our control tissues and therefore it seemed to be lung-specific. This epitope is of particular interest because a peptide derived from vimentin (amino acids 66-77), containing citrulline-71 and with leucine-70 replaced by alanine, binds with high affinity to HLA-DRB1*0401 MHC class II molecules, eliciting T cell activation [19]. In a more recent study, very similar vimentin-derived peptides (amino acids 59-78), also containing citrulline-71, bound to the same MHC class II molecules and to specific T cells in both RA and healthy individuals [20]. Furthermore, this peptide activated T cells in citrullinated vimentin peptide-immunised DR4 transgenic mice. Citrulline-71 is also part of an immunodominant B cell epitope [21], which was demonstrated, in a recent large study, to react with 37% of RA patients [3]. Our findings of ubiquitous expression of citrulline-71, together with T cell binding reported in a previous study [20] in both healthy people and patients with RA, makes it difficult to argue that this epitope is responsible for breaking tolerance leading to a disease-specific ACPA response.

Given that citrulline-71 in vimentin seems unlikely to lead to tolerance breakdown, the question then arises how does citrullination caused by inflammation break tolerance at all? One possible answer is that citrullination is not important, at least not in the initial stages of the generation of the ACPA response. Two studies have recently shown that patients with periodontitis, a known risk factor for RA, have a significantly elevated ACPA response, but strikingly this is not citrulline specific [22,23]. In a provisional report, we have found a similar pattern in patients with bronchiectasis [24]. The same pattern was also recently demonstrated by Brink *et al*. [25] where antibodies against arginine-containing peptides from RA autoantigens antedated development of citrulline-specific ACPA in serum samples taken from individuals years before they went on to develop symptomatic RA. The importance of uncitrullinated antigens in breaking tolerance, may well be supported by the fact that α-enolase was only found in its native, non-citrullinated form in lung tissues both from our study and in a previous report [18]. A mechanism for tolerance breakdown could be due to the fact that the immunodominant peptide of α-enolase (amino acids 5–21) comes from its most evolutionarily conserved region, including sequence homologies with bacterial enolases of >90% [26]. We have previously demonstrated cross-reactivity between human α-enolase and *Porphyromonas gingivalis* enolase [12] and it is highly likely that other bacterial enolases such as those expressed in the microbiome of the lung could have similar cross-reactivity leading to tolerance breakdown in the lung due to association with infective exacerbations of COPD or other chronic lung diseases.

Another possibility for tolerance breakdown in the lung are carbamylated peptides as they have been linked to inflammation and smoking [27], and can activate T cells in a MHC class II-dependent way [28]. Carbamylated lysine residues give rise to homocitrulline, which is structurally very similar to citrulline and is also recognised by ACPA and by the detection kit for citrullinated proteins (the AMC method) used in ours and other studies [29]. The detection of a homocitrulline residue in vimentin at position 104 in four out of six COPD samples and one out of six non-COPD may explain the apparent increase of citrullination shown by AMC in COPD in our study. More importantly, a higher expression of carbamylated peptides in pulmonary inflammation, such as COPD, could then break tolerance to citrullinated proteins, by acting as neoepitopes in the context of inflammation.

## Conclusions

We have shown that citrullination in the lung is highly abundant, with a modest increase induced by amplified inflammation in the context of COPD. To explain the citrulline specificity of autoimmunity in RA, we suggest a two-stage process in the evolution of the mature ACPA response. In an initial phase, antibodies to native proteins (such as enolase) may be induced by molecular mimicry between bacterial and self-antigens in susceptible individuals. Other mechanisms must be invoked for the early induction of antibodies to vimentin or fibrinogen, as neither have orthologues in bacteria. Epitope spreading to their citrullinated counterparts could be explained, in the case of vimentin, due to the ubiquitous expression of major citrullinated epitopes in the lung, resulting in the production of ACPA. Clearly, investigation of the two-stage hypothesis requires additional work, involving the evolution of very early autoantibody response in patients destined to get RA, through to the citrulline specificity of ACPAs, once the disease has evolved into its full clinical expression.

## Additional files

Additional file 1: Table S1. Characteristics of study subjects for lung tissue analysis. Additional file 2: Figures S1-S7. MS/MS spectra of seven vimentin peptides. Citrullination of vimentin [UniProt:P20152] in positions 71, 304, 346, 410 and 450, and homocitrullination in position 104. The MS/MS spectrum of tryptic peptides _70_ L(cit)SSVPGVR_78_, _71_(cit)SSVPGVR_78_, _295_FADLSEAAN(cit)NNDALR_310_, _344_QM(cit)EMEENFAVEAANYQDTIGR_365_, _403_LLEGEES(cit)ISLPLPNFSSLNLR_424_, _446_TVET(cit)DGQVINETSQHHDDLE_466_, _101_TNE(Hcit)VELQELNDRFANYIDKVR_122_ are shown, where cit refers to the citrullinated position and Hcit to the homocitrullinated position. The matched fragment ions of the y-type (red) and b-type (blue) are shown. The MS/MS spectrum in the bottom panel indicates matches of predominant fragment ions to the peptide sequence.

## Abbreviations

ACPA: Anti-citrullinated peptide/protein antibodies
AMC: Anti-modified citrulline
BAL: Bronchoalveolar lavage
CEP-1: Citrullinated α-enolase peptide 1
COPD: Chronic obstructive pulmonary disease
FDR: False discovery rate
GOLD: Global Initiative for Chronic Obstructive Lung Disease
HCD: High-energy collision dissociation
HRP: Horseradish peroxidase
kDa: KiloDaltons
PAD: Peptidylarginine deiminase
RA: Rheumatoid arthritis
