# Supplementary material for: Expression of citrulline and homocitrulline residues in the lungs of non-smokers and smokers: implications for autoimmunity in rheumatoid arthritis
Source: Arthritis Res Ther. 2015 Jan 20;17(1):9. doi: 10.1186/s13075-015-0520-x (PMC4349479; doi:10.1186/s13075-015-0520-x)
Supplement: Additional file 1: Table S1. — Characteristics of study subjects for lung tissue analysis. [file 13075_2015_520_MOESM1_ESM.pdf]

Additional File 1

Table S1: Characteristics of study subjects for lung tissue analysis

	never smokers	smokers	COPD current smokers	COPD ex-smokers
Number	10	10	13	8
Gender ratio (male/female)	4/6	6/4	13/0	8/0
Age (years)	56 (46-69)	56 (42-63)	65 (55-70)	72 (62-76)*§
BMI	25 (22-29)	23 (20-26)	24 (20-25)	26 (24-28)
Current-smoker / Ex-smoker	-	10/0	13/0	0/8
Smoking history (pack years)	0 (0-0)	33 (24-44)*	48 (33-62)*	45 (22-73)*
FEV1 post-bronchodilator (L)	2,5 (2,1-3,6)	2,9 (2,6-3,4)	2,6 (2,3-2,8)	2,4 (2,0-2,9)
FEV1 post-bronchodilator % predicted	100 (85-118)	103 (92-114)	75 (71-83)*§	75 (69-93)*§
FEV1 / FVC post-bronchodilator (%)	75 (72-81)	76 (74-84)	56 (53-63)*§	63 (57-67)*§
DLco (%)	89 (81-97)	83 (63-101)	64 (55-87)	89 (71-98)
Kco % predicted	100 (93-119)	91 (76-101)	73 (66-95)	94 (84-103)

FEV1 (forced expiratory volume in 1 second); FVC (forced vital capacity); DLco (carbon monoxide diffusing capacity); Kco (carbon monoxide gas transfer corrected for alveolar volume)

Data are presented as median (IQR)

Mann-Whitney U test: * P < 0,05 versus never smokers; § P < 0,05 versus smokers
