# Supplementary figures and images for: Expression of citrulline and homocitrulline residues in the lungs of non-smokers and smokers: implications for autoimmunity in rheumatoid arthritis
Source: Arthritis Res Ther. 2015 Jan 20;17(1):9. doi: 10.1186/s13075-015-0520-x (PMC4349479; doi:10.1186/s13075-015-0520-x)

## Additional File 2

Figure S1.

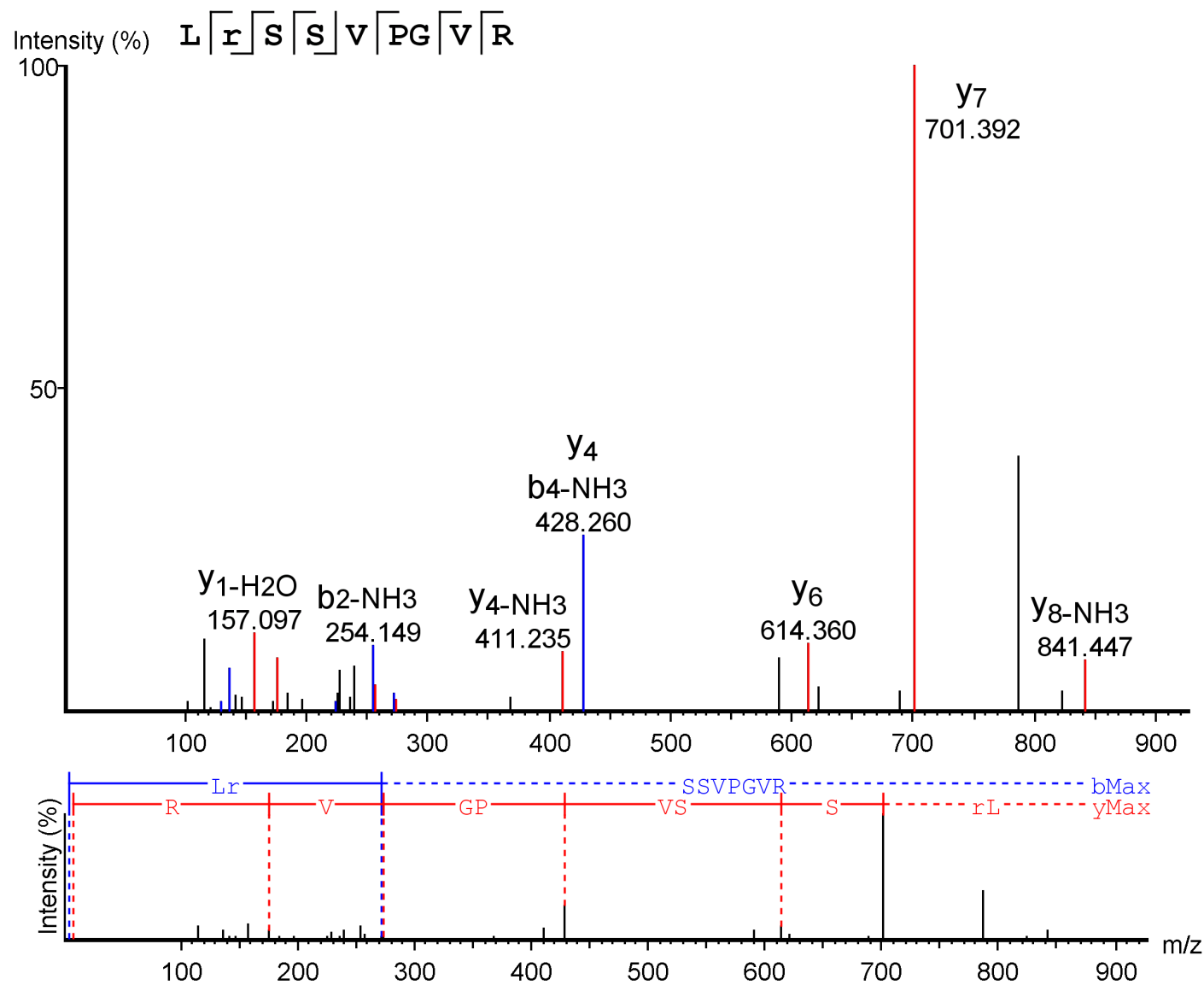

Figure S2.

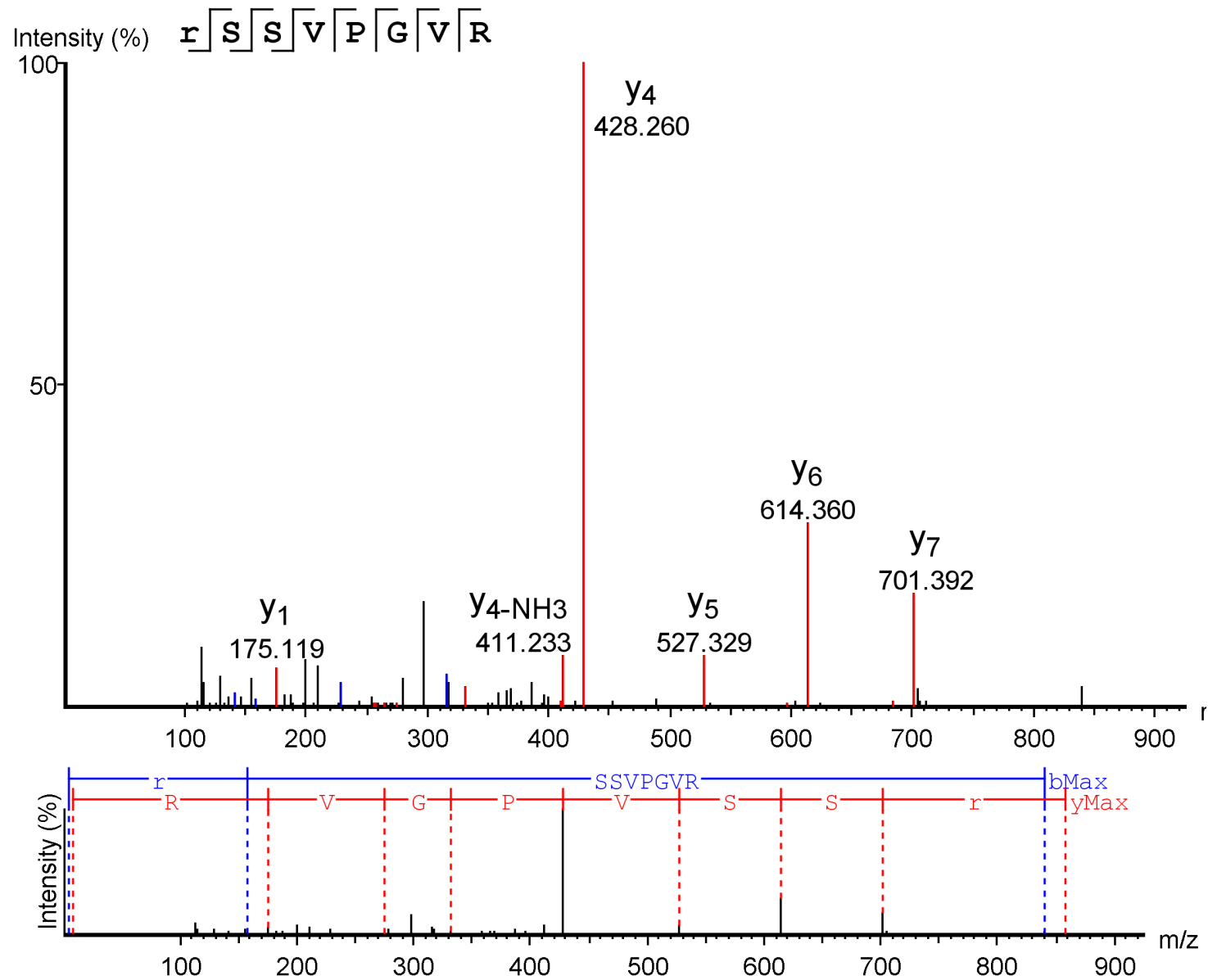

Figure S3.

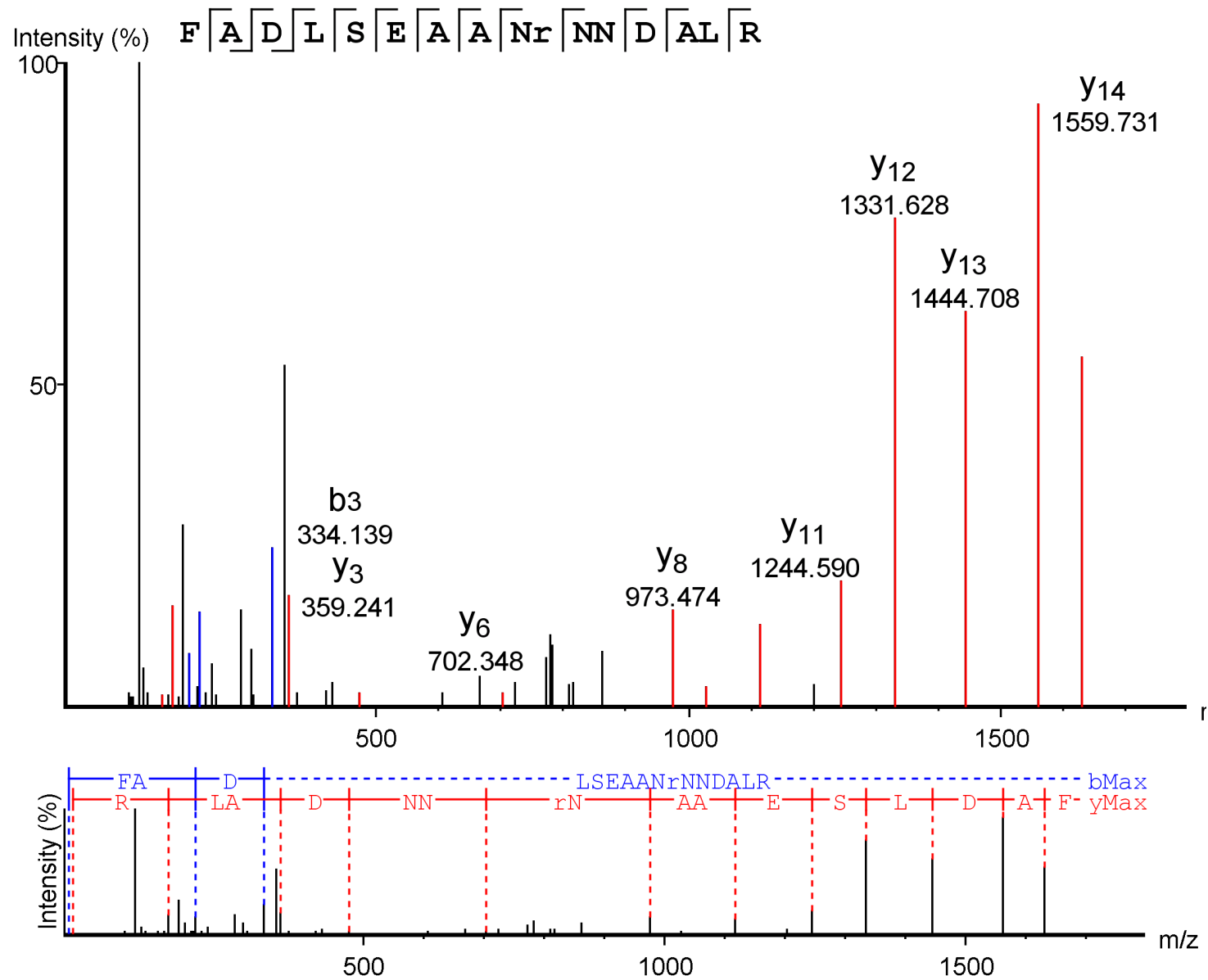

Figure S4.

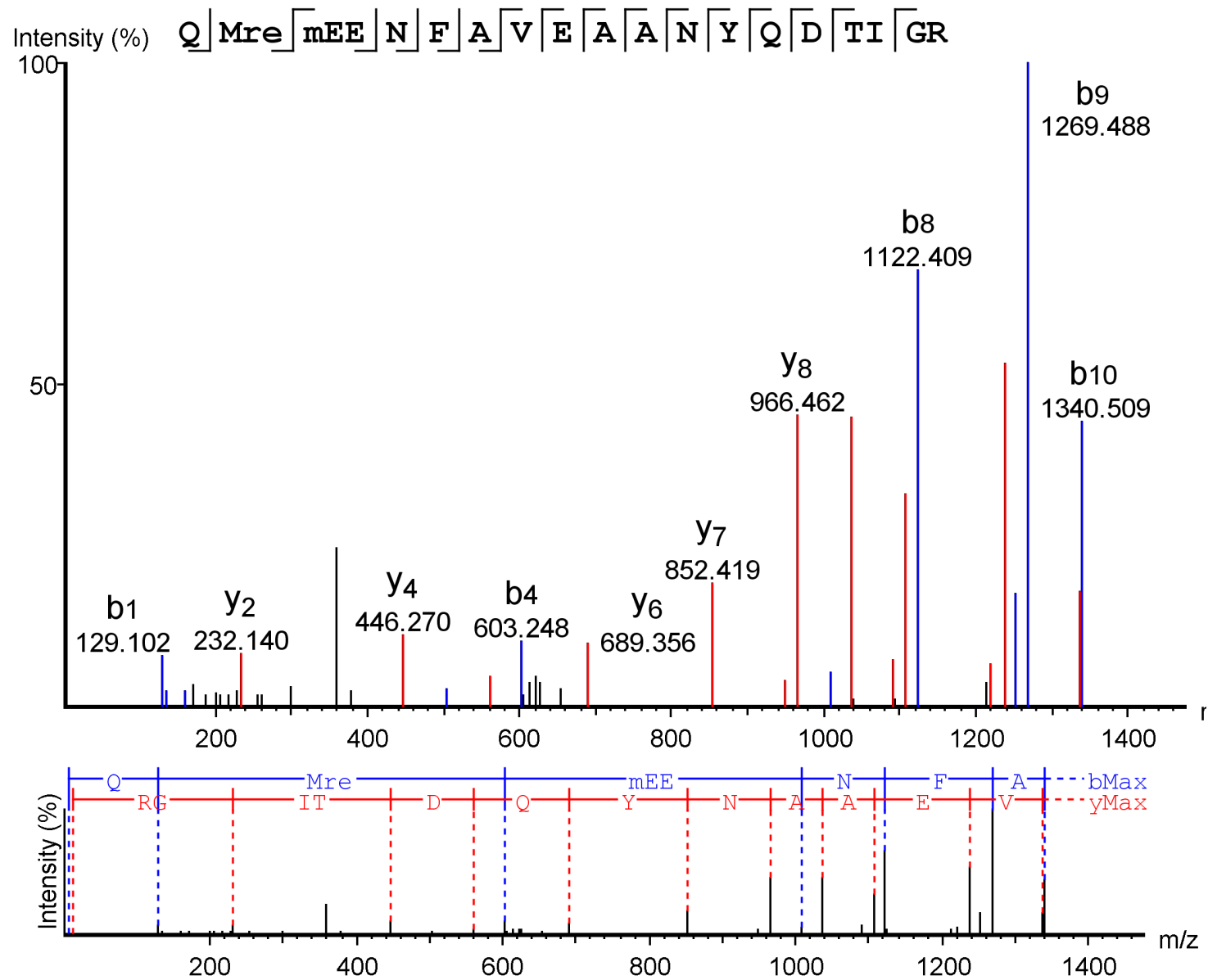

Figure S5.

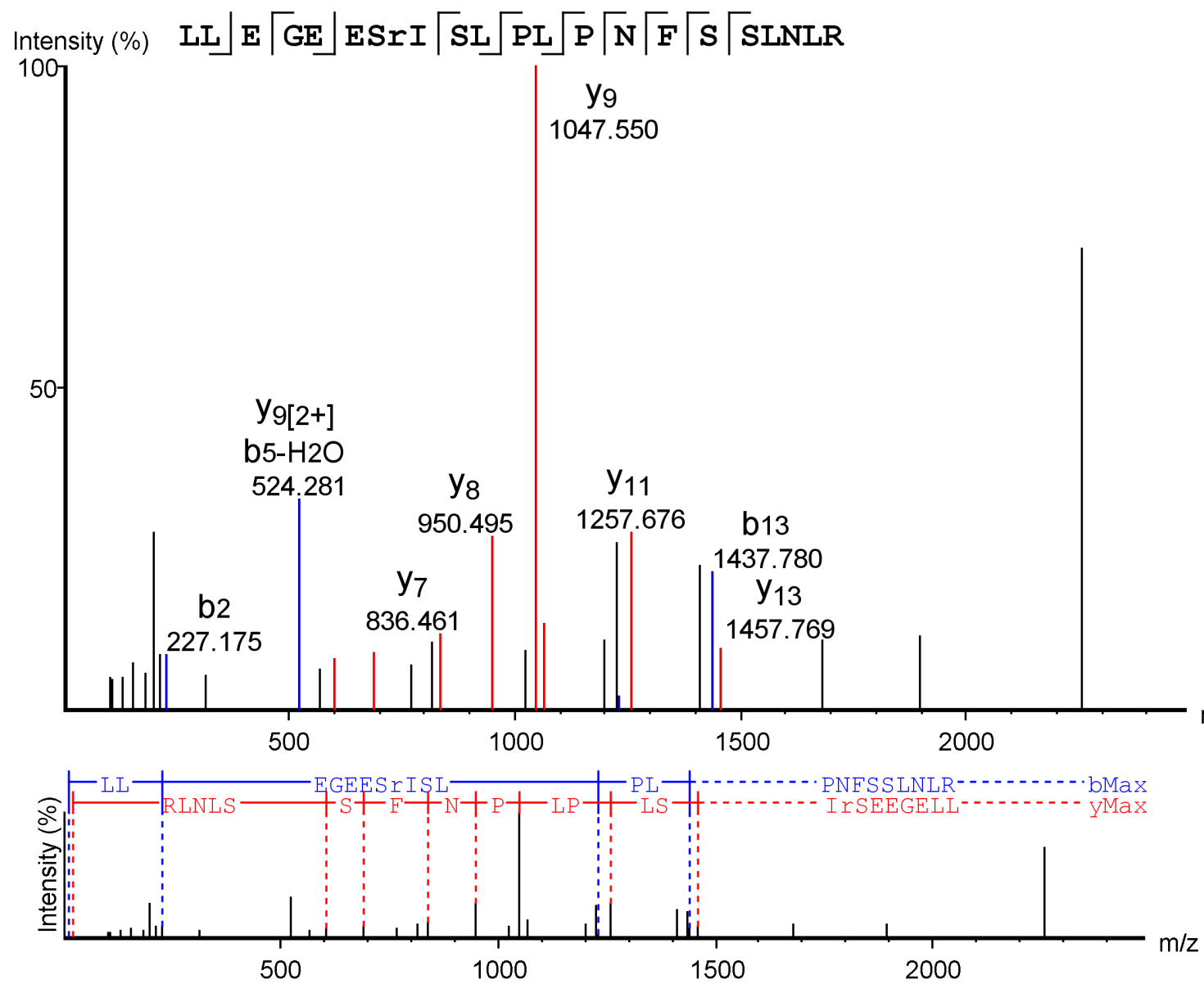

Figure S6.

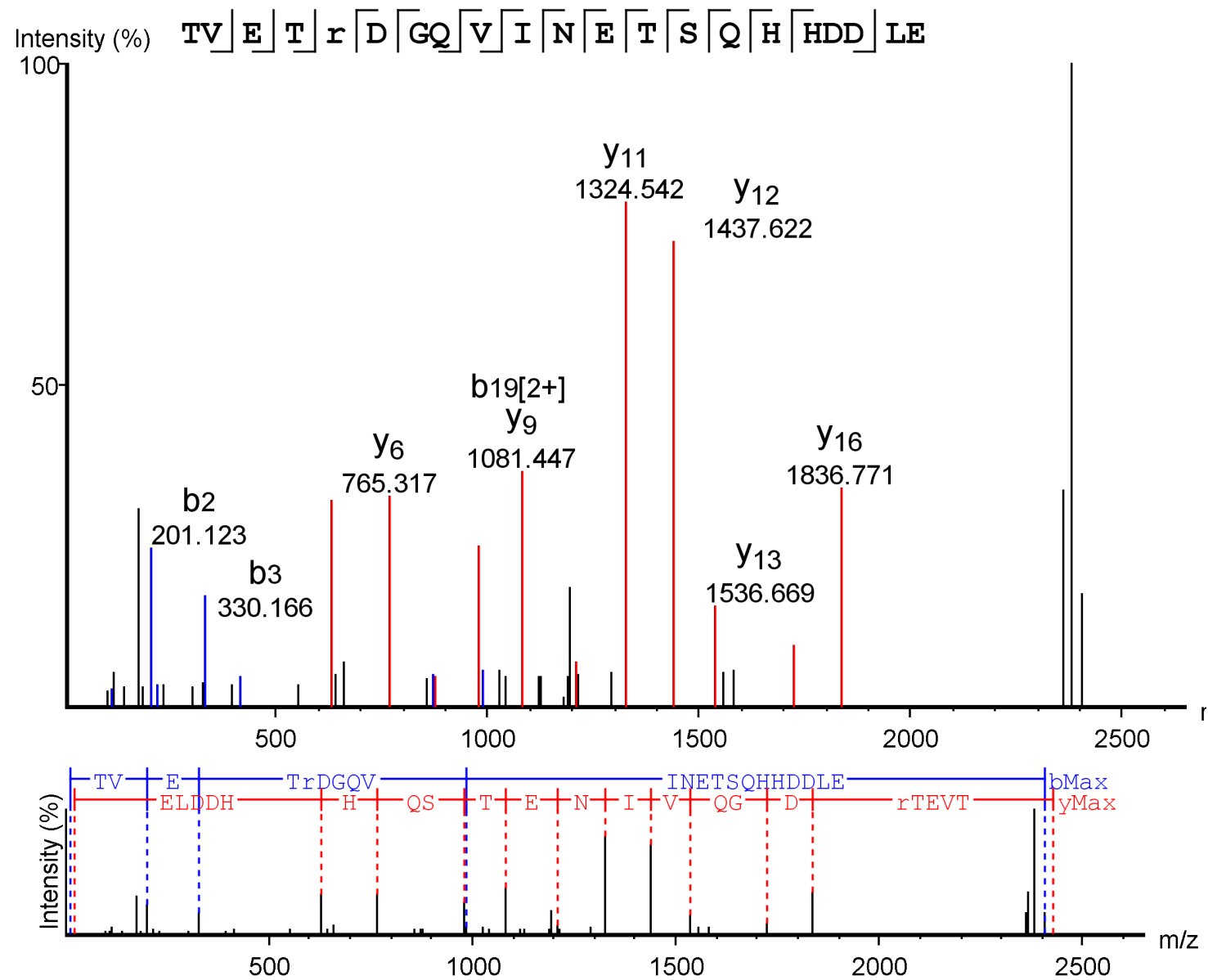

Figure S7.

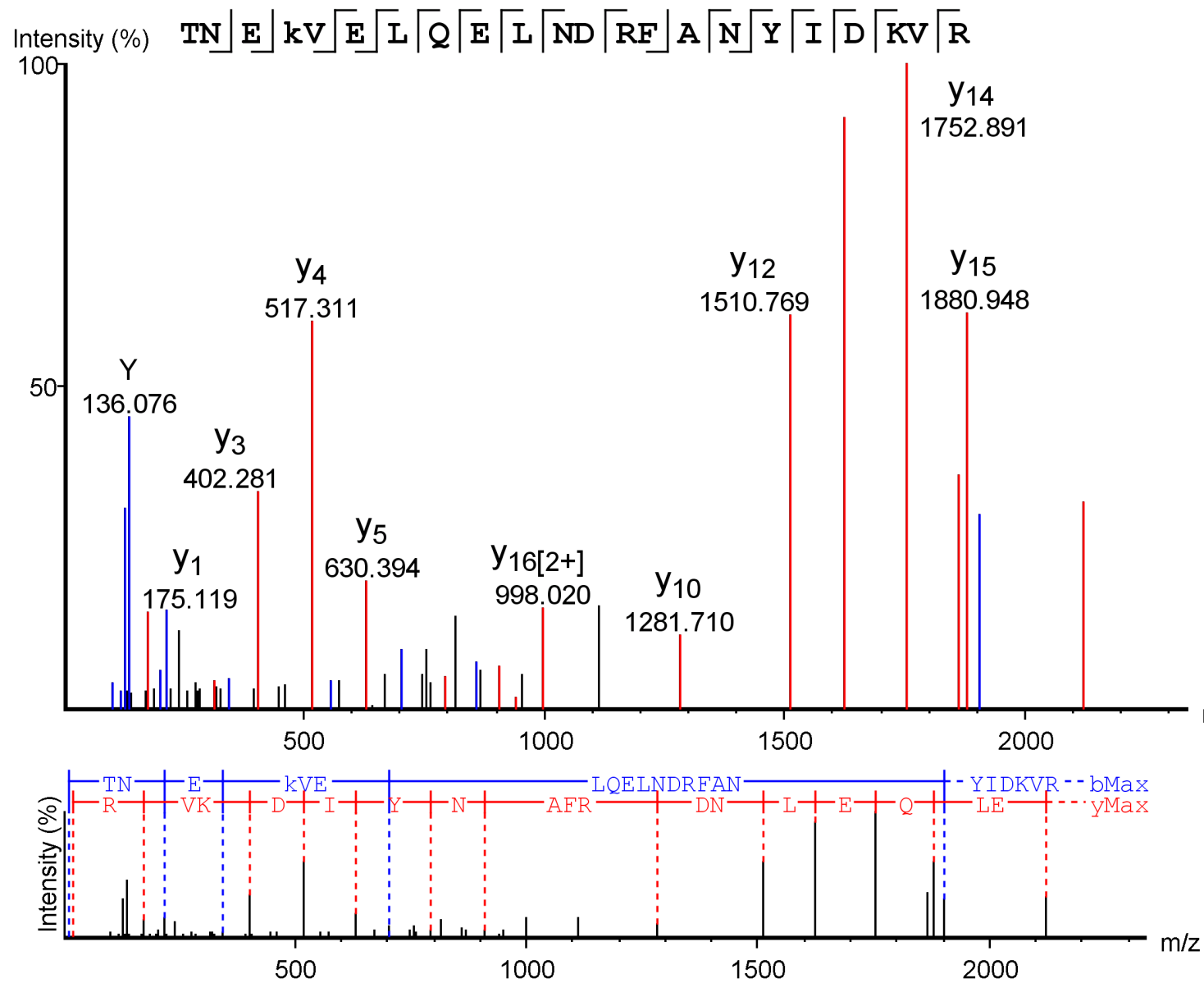

Supplement: Additional file 2: Figures S1-S7. — MS/MS spectra of seven vimentin peptides. Citrullination of vimentin [UniProt:P20152] in positions 71, 304, 346, 410 and 450, and homocitrullination in position 104. The MS/MS spectrum of tryptic peptides 70 L(cit)SSVPGVR78, 71(cit)SSVPGVR78, 295FADLSEAAN(cit)NNDALR310, 344QM(cit)EMEENFAVEAANYQDTIGR365, 403LLEGEES(cit)ISLPLPNFSSLNLR424, 446TVET(cit)DGQVINETSQHHDDLE466, 101TNE(Hcit)VELQELNDRFANYIDKVR122 are shown, where cit refers to the citrullinated position and Hcit to the homocitrullinated position. The matched fragment ions of the y-type (red) and b-type (blue) are shown. The MS/MS spectrum in the bottom panel indicates matches of predominant fragment ions to the peptide sequence. [file 13075_2015_520_MOESM2_ESM.pdf]
